# *Candidatus* Sodalis melophagi sp. nov.: Phylogenetically Independent Comparative Model to the Tsetse Fly Symbiont *Sodalis glossinidius*

**DOI:** 10.1371/journal.pone.0040354

**Published:** 2012-07-17

**Authors:** Tomáš Chrudimský, Filip Husník, Eva Nováková, Václav Hypša

**Affiliations:** 1 Faculty of Science, University of South Bohemia, České Budějovice, Czech Republic; 2 Institute of Parasitology, Biology Centre, Academy of Sciences of the Czech Republic, České Budějovice, Czech Republic; Loyola University Medical Center, United States of America

## Abstract

Bacteria of the genus *Sodalis* live in symbiosis with various groups of insects. The best known member of this group, a secondary symbiont of tsetse flies *Sodalis glossinidius*, has become one of the most important models in investigating establishment and evolution of insect-bacteria symbiosis. It represents a bacterium in the early/intermediate state of the transition towards symbiosis, which allows for exploring such interesting topics as: usage of secretory systems for entering the host cell, tempo of the genome modification, and metabolic interaction with a coexisting primary symbiont. In this study, we describe a new *Sodalis* species which could provide a useful comparative model to the tsetse symbiont. It lives in association with *Melophagus ovinus*, an insect related to tsetse flies, and resembles *S. glossinidius* in several important traits. Similar to *S. glossinidius*, it cohabits the host with another symbiotic bacterium, the bacteriome-harbored primary symbiont of the genus *Arsenophonus*. As a typical secondary symbiont, *Candidatus* Sodalis melophagi infects various host tissues, including bacteriome. We provide basic morphological and molecular characteristics of the symbiont and show that these traits also correspond to the early/intermediate state of the evolution towards symbiosis. Particularly, we demonstrate the ability of the bacterium to live in insect cell culture as well as in cell-free medium. We also provide basic characteristics of type three secretion system and using three reference sequences (16 S rDNA, *groEL* and *spaPQR* region) we show that the bacterium branched within the genus *Sodalis*, but originated independently of the two previously described symbionts of hippoboscoids. We propose the name *Candidatus* Sodalis melophagi for this new bacterium.

## Introduction

The genus *Sodalis* belongs to the symbiotic bacterial lineages that adopted several different types of symbiosis with their hosts, ranging from facultative commensals to obligate mutualists [1], [2]. *Sodalis* and closely related bacteria were described from a broad spectrum of insect hosts including tsetse flies [3], weevils [4]–[7], chewing lice [8], hippoboscid louse flies [9], ants [10], scale insects [11], aphids [12], stinkbugs [13], [14], and cerambycid beetles [15]. Also, some “secondary” symbionts of psyllids and mealybugs cluster with the *Sodalis* clade [16], [17]. Within symbiotic Enterobacteriaceae, diversity of the *Sodalis* clade is comparable only with the genus *Arsenophonus* [18].

The first described, best known and most frequently investigated member of the genus is *S. glossinidius*, S-symbiont of tsetse flies [3]. Its significance for the host is still not clear, but a possible influence on the host longevity and resistance to trypanosomes has previously been suggested [19]. Several molecular analyses and genetic experiments made *S. glossinidius* an important model for investigating evolution and biology of symbiotic bacteria [20]–[22]. It has been employed in research of various biological traits, such as the structure and role of secretion systems [21], the function of the iron acquisition system [23] or the usage of the quorum sensing system [24]. Moreover, *S. glossinidius* proved to be among the few symbionts that could be maintained in *in vitro* culture in insect cells as well as in the cell-free media [3], [25]. This feature has been attributed to the initial or intermediate state of the *S. glossinidius* shift towards symbiosis. One of the most frequently discussed topics in this respect is the state and function of the type three secretion system (TTSS) in this bacterium. Three different copies of the TTSS (SSR-1, SSR-2 and SSR-3) has been detected in *S. glossinidius* [26], [27] and a possible role of the SSR-2 in invading host cells has been proposed [21].

Within the host, *S. glossinidius* constitutes part of a complex bacterial community which also contains P-symbiont *Wigglesworthia glossinidia* [28] and alphaproteobacterium *Wolbachia* [29], [30]. Recent investigations show that the whole community may be even richer and contain an array of other bacteria [31]. Such complex host-symbiont systems provide a unique opportunity for comparing genomes in different states/modes of symbiosis and studying processes of their metabolic complementation [32], [33]. For the *Sodalis-Wigglesworthia*-*Glossina* association, complete genomes of both bacteria have been sequenced and annotated [26], [34]. Even though each of the sequenced genomes came from different host species, an interaction between *Sodalis* and W*igglesworthia* via thiamine synthesis could be detected by their comparison [27] and this view was further corroborated by an experimental approach [35] and sequencing of *Wigglesworthia* lineage from *Glossina morsitans* [36].

An establishment of a more complete picture of *Sodalis* genome evolution will require complete genome sequences for a more diverse array of *Sodalis* isolates, and of similar complex systems involving this bacterium. Although tsetse flies are the most important blood feeding brachycerans, there are several related groups of dipterans that display many similar features such as the feeding strategy, vivipary and transmission of trypanosomes.

A member of the genus *Sodalis*, phylogenetically independent on the tsetse symbiont, has already been described from hippoboscoid species *Craterina melbae* [9]. Here, we characterize a new member of the *Sodalis* lineage, inhabiting gut and other tissues of another hippoboscoid, *Melophagus ovinus*. The presence of several symbiotic bacteria in this species has long been known. According to morphological investigations of several researchers summarized by Paul Buchner [37], *Melophagus ovinus* contains symbiotic bacteria within enlarged epithelial cells of a specialized section of the midgut (bacteriome). This P-symbiont was recently characterized by molecular techniques as a member of *Arsenophonus* clade and is likely to play a role resembling that of *Wigglesworthia* in tsetse flies (Nováková et al., in prep). In addition, some of the authors recognized two other bacteria in the sheep keds. The first is *Bartonella melophagi* (originally described as *Rickettsia melophagi* and *Wolbachia melophagi*), which is localized extracellularly along microvilli of the midgut. The second type of bacterium described in Paul Buchner’s work resembles *Candidatus* Sodalis melophagi as presented in this study: *“In the low zones of the midgut epithelium…, there are additional delicate bacteria, sometimes forming rather long filaments, which also must not be confused with the symbionts.”* The whole system thus remarkably resembles the *Wigglesworthia*-*Sodalis* association in tsetse flies and can provide important data for a comparative study. In this study, we present a basic molecular and morphological characterization of the new *Sodalis* linage and overview the composition of its TTSS. We suggest the new name *Candidatus* Sodalis melophagi for this bacterium and extend the available *Sodalis* spectrum with three additional samples which allow for more precise phylogenetic characterization.

## Results

### Sequence Data

Sequences obtained by PCR for the investigated samples and their accession numbers are summarized in Table S1. The 16 S rDNA sequence from *Candidatus* Sodalis melophagi sp. nov. displayed 98.48% similarity to 16 S rDNA of *S. glossinidius*. Illumina assemblies produced preliminary draft sequences from which only selected gene regions were used here for the formal description and basic phylogenetic characterization. These regions, including all TTSS genes (Table S1) and *groEL* chaperonin, were of a high quality and did not contain any SNPs. In order to avoid assembly artifacts affecting the sequence accuracy of highly similar paralogous regions, a partial sequence for 16 S rRNA gene was obtained through Sanger sequencing as described above and was used for inferring phylogeny. Sanger sequenced *groEL* partial sequence was identical to the sequence acquired from Illumina data and we therefore used the full length *groEL* for phylogenetic reconstruction.

### Type Three Secretion System (TTSS)

**Figure 1:**
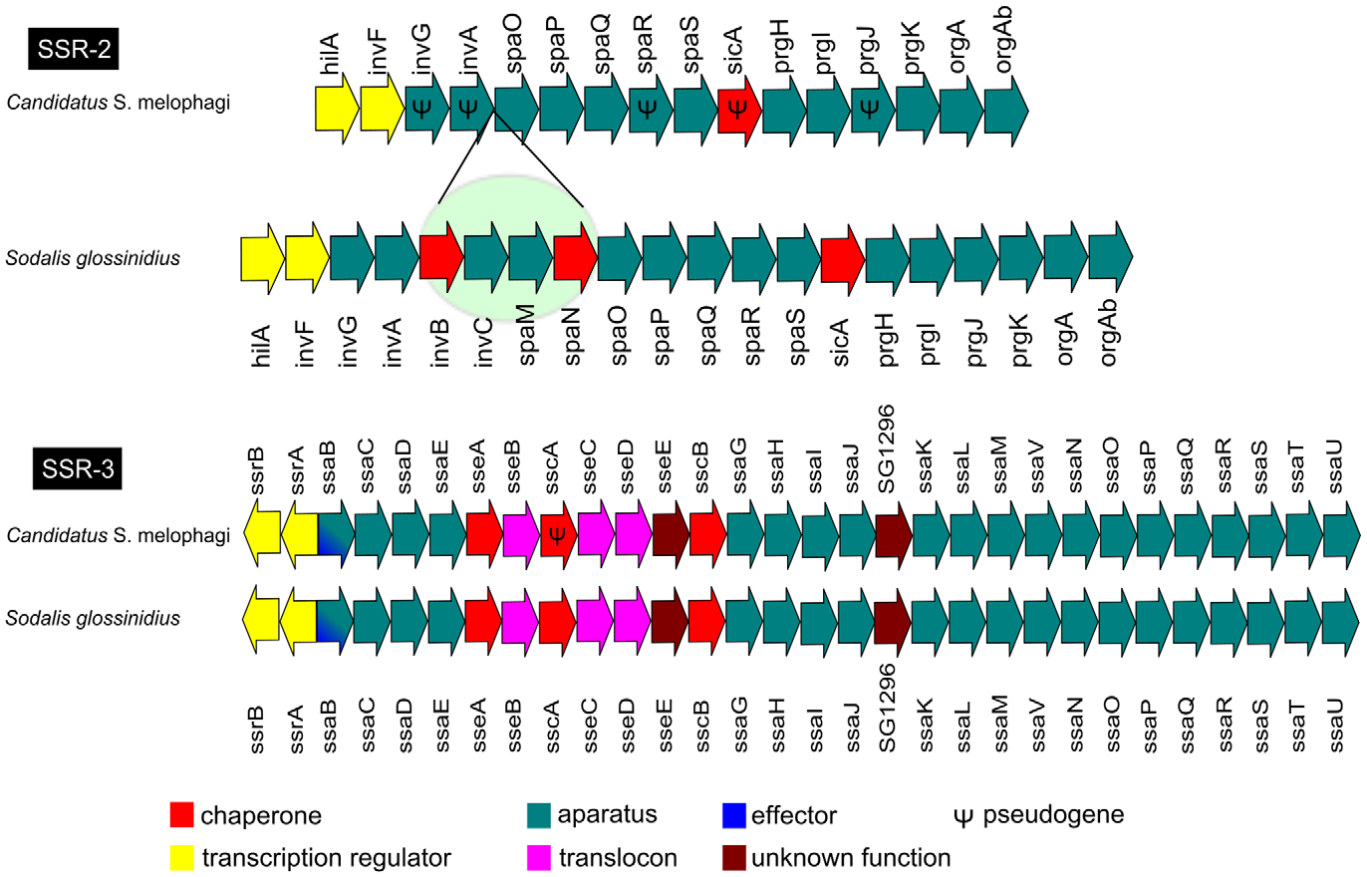
Composition of SSR-2 and SSR-3 copies of *Candidatus* Sodalis melophagi TTSS. Ψ – putative pseudogene (see Material and Methods).

*Candidatus* Sodalis melophagi possesses only SSR-2 and SSR-3 copies of the TTSS in its genome, while SSR-1 is completely missing. Extensive BLASTX searches did not produce any significant hits for *S. glossinidius* SSR-1 either in Illumina contigs or raw reads. The gene order and content of SSR-2 and SSR-3 of *Candidatus* Sodalis melophagi is very similar, but not identical to that in *S. glossinidius* (Figure 1). The SSR-2 sequence comprises 11 protein coding genes: *orgAbA*, *prgKIH*, *spaSQPO*, *invF* and *hilA*; and 5 pseudogenes: *prgJ*, sicA, spaR and *invAG*. In comparison with *S. glossinidius*, it lacks 4 genes: *invB,* *invC* and *spaLMN*. The SSR-3 comprises 29 protein coding genes: *ssrAB*, *ssaBCDEGHIJKLMNOPQRSTUV*, *sseABCDE*, *sscB*, a protein similar to locus SG1296 of *S. glossinidius*, and a single pseudogene: *sscA*. Sequences of *Candidatus* Sodalis melophagi TTSS genes were deposited in GenBank as a part of two annotated contigs for each of the islands (Table S1).

### Phylogenetic Analyses

**Figure 2:**
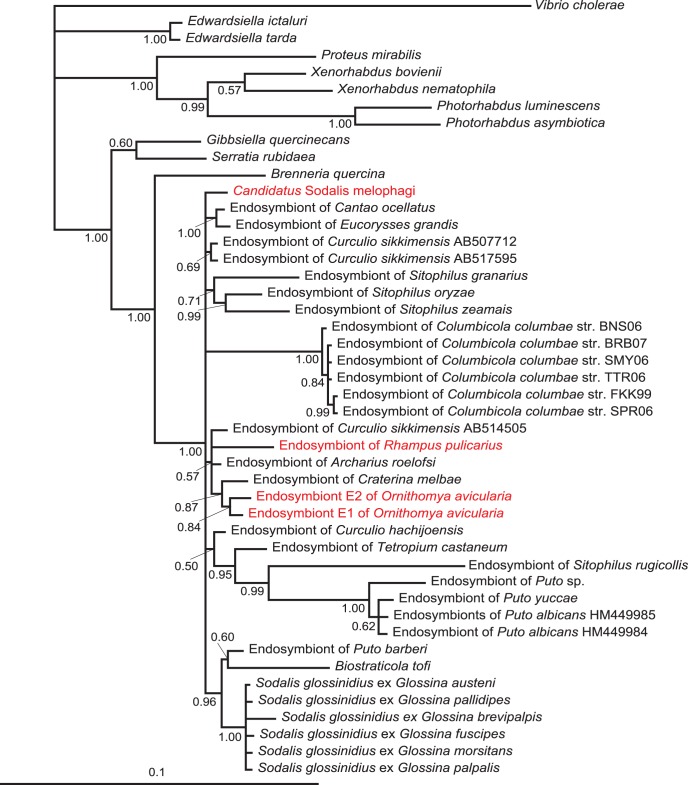
16 S rDNA tree derived by BI analysis in MrBayes. Posterior probabilities are indicated by the numbers at the nodes. New *Sodalis* lineages added in this study are printed in red.

**Figure 3:**
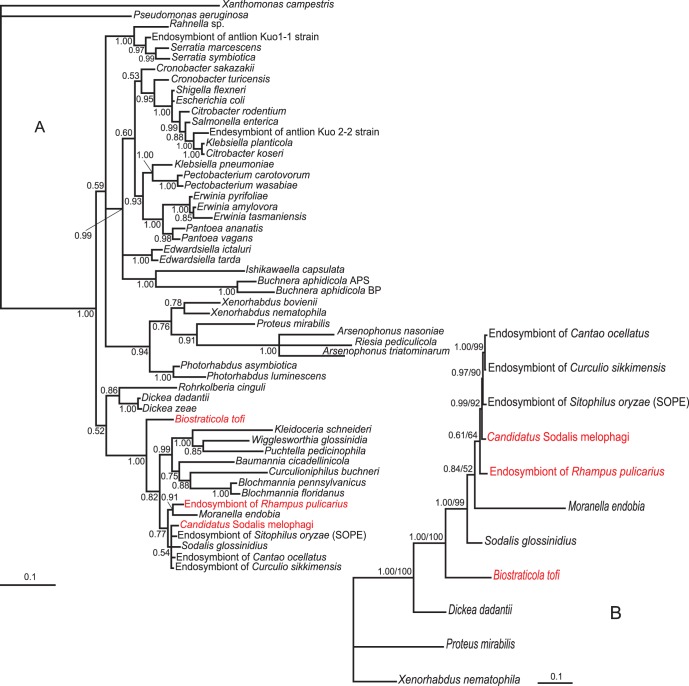
Phylogenetic trees derived from *groEL* matrices by BI in MrBayes. New *Sodalis* lineages added in this study are printed in red. **A**: The tree inferred from aminoacid matrix. Posterior probabilities are indicated by the numbers at the nodes. **B**: The tree inferred from nucleotide matrix restricted taxonomically to the *Sodalis* branch. The numbers at the nodes show the posterior probabilities and bootstrap values from the identical topology obtained by ML in PhyML.

**Figure 4:**
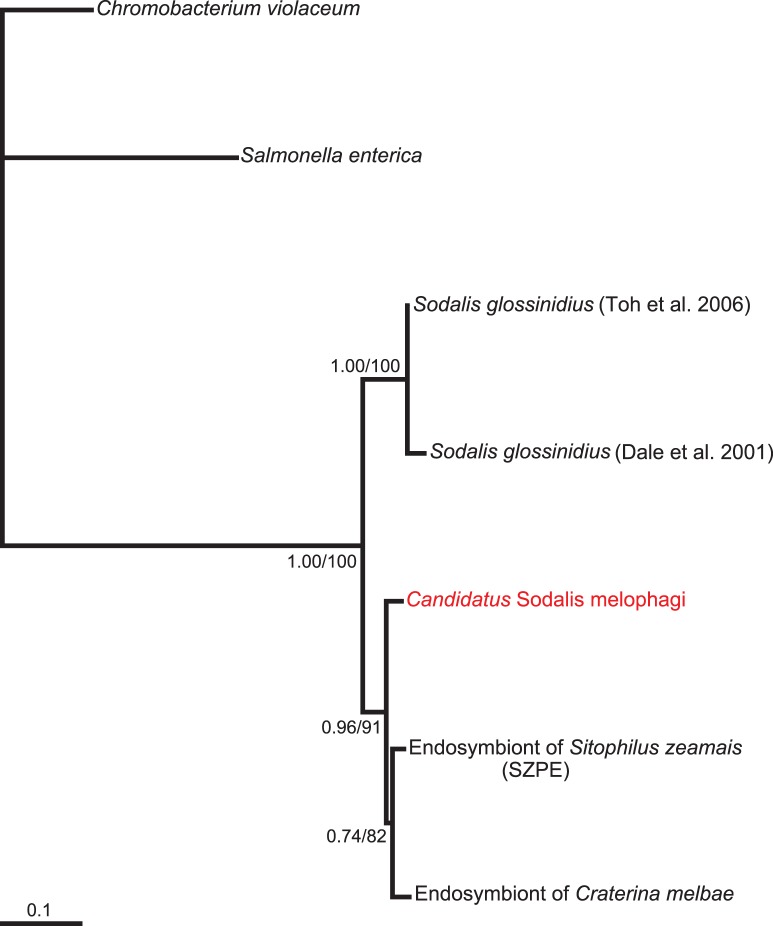
Phylogenetic tree derived from *spaPQR* region by BI in MrBayes. The numbers at the nodes show the posterior probabilites and bootstrap values from the identical topology obtained by ML in PhyML. New *Sodalis* lineages added in this study are printed in red.

The lengths of individual matrices and numbers of variable positions are summarized in Table S2. All phylogenetic trees clearly indicate that the novel bacterium belongs to the genus *Sodalis* (Figures 2, 3, 4, Figure S1, Figure S2). In the trees derived from 16 S rRNA gene sequence data and amino acid sequence of *groEL*, *Candidatus* Sodalis melophagi clusters within a large polytomy and its precise position within the genus is thus uncertain (Figures 2–3A, Figure S1, Figure S2). However, even this unresolved topology excludes its relationship to any of the two other hippoboscoid-derived *Sodalis* members. This conclusion is further supported by the nucleotide matrix for *groEL* (Figure 3B) with a reduced sampling and the *spaPQR* concatenate (Figure 4). Although the exact topology slightly varies with the methods and parameters of the analysis, the three hippoboscoid lineages always form a polyphyletic/paraphyletic assemblage.

### *In vitro* Culture

Bacterial colonies were clearly visible after 8 days of cultivation. Colonies were white, raised, and circular with entire edges. Their size was irregular ranging from 0.5 to 1 mm. The variable size of the colonies was almost certainly not due to new mutations, since subculturing on fresh plates yielded the same variability for each individual colony. The irregular colony morphology was described also in case of a type strain M1^T^ of the *Sodalis glossinidius* [3] and is probably population dependent as is the case in other microaerophilic bacteria [38].

The type strain CZ^T^ was established by isolation of a single bacterial colony and was used for C6/36 cells infection. In the C6/36 cell culture, bacteria were predominantly attached to cell surface or free in the medium, but they were also observed inside the cells. Genomic DNA purified from bacterial colonies of *Candidatus* Sodalis melophagi CZ^T^ was used as a template for Illumina mate pair sequencing.

### Microscopy

**Figure 5:**
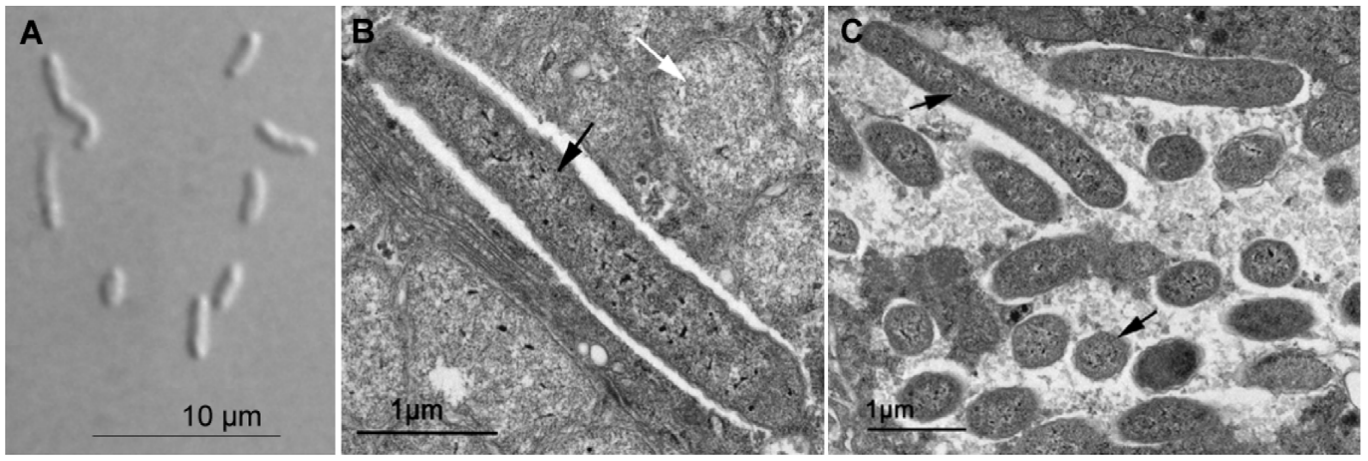
Morphology and ultrastructure of *Candidatus* Sodalis melophagi. **A**: *In vitro* cell culture in Nomarski contrast. **B**, **C**: Cells of *Candidatus* Sodalis melophagi in bacteriome. Black arrows – cells of *Candidatus* Sodalis melophagi, white arrows – cells of the primary endosymbiont of the genus *Arsenophonus*.

Under electron microscopy, *Sodalis* cells within the host tissue corresponded well to their light microscope characteristics (Figure 5A). They appeared as rods reaching from approx. 1 to 4 μm, depending on the angle of the section, and were located mainly at the periphery of the bacteriome, sometimes in close association with the P-symbionts (Figures 5B,C).

### Description of *Candidatus* Sodalis Melophagi sp. nov

*Candidatus* Sodalis melophagi (me.lo.phag.í; *melophagi* of the genus *Melophagus*).

*Candidatus* Sodalis melophagi is a Gram-negative bacterium isolated from the sheep ked *Melophagus ovinus*. The bacteria were detected in heamolymph and bacteriome. The cells are rod-shaped non-motile and non-spore forming under laboratory conditions. The average length is 1.9 μm, however, bacteria ranging from 0.6 to 5 μm in length were observed. The bacteria grow intracelullarly and extracellularly in presence of *Aedes albopictus* cell line C6/36 under aerobic conditions. They can also be cultivated axenically both in liquid and solid media containing enzymatically digested proteins as a source of nitrogen, with the addition of blood under the microaerophilic atmosphere (5% O_2_ balanced with carbon dioxide) at 27°C.

## Discussion

Remarkably, the main biological traits of *Candidatus* Sodalis melophagi and its relation to the host resemble those of *Sodalis glossinidus* in tsetse flies. In both cases the hosts are exclusively blood feeding organisms reproducing by vivipary and their *Sodalis* plays a role of an “accessory” symbiont accompanying phylogenetically distant P-symbiont (*Wigglesworthia* in tsetse flies and *Arsenophonus* in *Melophagus*). This status of *Candidatus* Sodalis melophagi is strongly indicated by phylogenetic characteristics as well as location and morphology revealed by electron microscopy. Similar to *S. glossinidius*, the rod-shaped cells of *Candidatus* Sodalis melophagi are not restricted to the bacteriocytes but they infect various non-specialized cells and can be cultivated from haemolyph. It is therefore interesting to note that according to phylogenetic analyses its symbiosis with the host originated independently of the *S. glossinidius* as well as of the third hipoboscoid lineage, the symbiont of *Craterina melbae*. Although exact position of *Candidatus* Sodalis melophagi within the *Sodalis* clade varies with method and taxon sampling, it never clusters with any of the two other hippoboscoid-derived lineages. Within the *Sodalis* clade, such host-symbiont incongruence is not unique to the hippoboscoid-derived lineages. It is also expressed by the distribution of several other samples, particularly those from coleopteran and homopteran hosts (Figure 2, Figure S1) and clearly demonstrates the capability of *Sodalis* to spread by horizontal transfers. In contrast, the close relationships of the two new 16 S rDNA sequences obtained from *Ornithomya* with the *Sodalis* previously described from *Craterina melbae* suggests that these two hippoboscoid genera may share the same symbiotic lineage inherited from their common ancestor. However, the sampling of *Sodalis* lineages available from other insect groups is highly incomplete. In addition, the position of some sequences, such as *Sodalis* from *Sitophilus rugicollis* is affected by long-branch attraction due to the high AT content; instability of this lineage has been found in previous analyses [9]. Thus, although we extended the sampling with additional three sequences, any interpretations of the modes and mechanism of *Sodalis* transmission can under the current circumstances be only working hypotheses rather than serious conclusions based on the data.

While the draft genomic data of *Candidatus* Sodalis melophagi is currently under investigation, we preliminarily analyzed the composition and structure of the TTSS. Significance of this system in symbiosis evolution has previously been suggested [39] and investigated in several symbiotic lineages [21], [40]–[43]. These genes are also among the few sequences that are currently available and can be compared between different *Sodalis* taxa. In *S. glossinidius*, experimental work indicated that TTSS of SPI1 type from *Salmonella* (later designated as SSR-2) is essential for entering the host cell. Presence and apparent functionality of this system was subsequently confirmed in another *Sodalis*, the primary endosymbiont of *Sitophilus zeamais* (SZPE) [42]. However, further studies revealed another two copies of TTSS in *S. glossinidius* genome, SSR-1 closely related to the Ysa system of *Yersinia*, and SSR-3 related to *Salmonella* SPI2 [26], [27], [40]. The secretion systems in *Candidatus* Sodalis melophagi show clear similarity to *S. glossinidius* genes but the whole machinery is much less complex; SSR-1 is completely missing and SSR-2 is highly eroded. However, the form corresponding to the SSR-3 is complete and possibly functional. Its structure and gene content is highly similar to that in *S. glossinidius*, except for a pseudogenized state of *sscA*, encoding a putative chaperone of secreted protein SseC [44].

The differences between TTSS in *S. glossinidius* and *Candidatus* Sodalis melophagi pose an interesting question about the origin, role and significance of TTSS and its different copies in the genus *Sodalis*. As the two compared lineages, *Candidatus* Sodalis melophagi and *S. glossinidius* are not closely related and the whole *Sodalis* tree is currently undersampled, it is impossible to hypothesize whether SSR-1 was lost in the former one or acquired by the latter one after diversification of their ancestor. The significance of TTSS in the two lineages is even more difficult to assess. In *Candidatus* Sodalis melophagi, the SSR-3 alone or possibly in synergy with the retained functional genes of SSR-2, seems to be sufficient for maintenance of the intracellular lifestyle. This view, however, is based solely on the observed presence/absence of the genes and their comparison to the experimental results from *S. glossinidius* [21]. It should be taken into account that other related systems, such as flagellar export apparatus, can participate in the host cell invasion. Despite the knockout experiments, the situation is similarly unclear in *S. glossinidius* where the presence of SSR-3 has not been known at the time of the experimental work and its significance could not be investigated. From the genomic point of view, *S. glossinidius* has so far been the only member of the genus for which a detailed characterization of the genome and some metabolic capabilities is available. The quality of *Candidatus* Sodalis melophagi paired-end data retrieved from the bacteriome sequencing and additional mate pair data recently obtained from the pure culture suggest that a draft genome of this symbiont could be established and used for further comparisons. Such analysis, comparing reduction, structure, and possible adaptive changes of independent but closely related bacteria from two hosts with similar but unique biology will provide important insight into the symbiogenetic processes. In respect to the future work, it may help to discriminate between the random and symbiosis-associated modifications and indicate candidate genes for a more detailed investigation.

## Materials and Methods

### Ethic Statement

All field studies did not involve protected or endangered organisms. They were not performed on privately-owned or protected locations and were performed according to the law of the Czech Republic.

### Insect Samples, DNA Extraction, Sequencing and Data Assembly

Adults of *Melophagus ovinus* were obtained commercially from a licensed family sheep farm at Krásetín, Czech Republic. A midgut region with the bacteriome was dissected into the phosphate buffered saline (PBS) and total genomic DNA was extracted from each single adult by QIAamp DNA Micro Kit (Qiagen). DNA concentration was determined using NanoDrop2000 (Thermo Scientific) and its quality was assessed in 2% agarose gel using standard electrophoresis. PCR was carried out as described previously [18] with bacterial primers for 16 S rDNA and *groEL* (Table S3). The same procedure was followed for three additional samples. Two of them were *Sodalis* bacteria from other insect hosts, *Ornithomya avicularia* (Diptera, Hippoboscidae) and *Rhampus pulicarius* (Coleoptera, Curculionidae), and the third was *Biostraticola tofi* strain BF36^T^, a free-living bacterium supposedly closely related to *Sodalis* [45]. Laboratory culture of *B. tofi* was obtained from the DSMZ microorganism collection (Germany), *R.* *pulicarius* was collected near České Budějovice (Czech Republic); according to the law of the Czech Republic, no permits are required for collection of this organism. Samples of *O. avicularia* were provided by Department of Zoology (University of South Bohemia); the collections were done during the ornithological research performed in accordance with the law of the Czech Republic.

We used 3 μg of genomic DNA isolated from *M. ovinus* bacteriome as a template for an Illumina paired-end library with an insert size of 300 bp. Library construction and sequencing on one lane in a 100 bp run was carried out at Keck Microarray Resource, West Haven CT, USA. Reads obtained underwent adaptor and quality trimming, and were further processed using two different approaches. First, the reads with significant BLASTN [46] hit to *Sodalis glossinidius* genome sequence (NC_007712) were filtered and the retrieved subset was assembled using CLC Genomic Workbench (CLC bio A/S) with parameters set to the following values: similarity 0.9, length fraction 0.9, costs for deletion/insertion/mismatch 3. Second, de-novo assembly of all the processed reads into contigs was done on CLC Genomic Workbench (CLC bio A/S) under the same parameter setting. Contigs from this assembly were binned based on their average coverage and BlastX [46] hits against available bacterial genomes. Since some of the sequences were used in this study for phylogenetic reconstruction, we checked the accuracy of the Illumina-derived sequences by an independent Sanger sequencing of the *groEL* gene.

### Type Three Secretion System (TTSS) Annotation

Contigs spanning corresponding genes to TTSS islands from *Sodalis glossinidius* were retrieved based on the Blast X [46] results from both assemblies. In order to obtain a single contig for each of the TTSS islands, gaps were closed using targeted Sanger sequencing (Table S3). Mapped sequences were checked for presence of single nucleotide polymorphisms (SNPs). ORF prediction was done using CLC Genomic Workbench (CLC bio A/S) with the minimum length set to 30 AA. Gene annotation based on similarity confirmed by BLAST searches was performed manually in the same software. All genes that contained frame shift mutation or stop mutations were tentatively classified as pseudogenes. The *hilA* previously classified as pseudogene [26], [27] was annotated as functional based on recent experimental work [47].

### Phylogenetic Analyses

We used three different regions for the phylogenetic reconstruction, 16 S rDNA, *groEL* and the TTSS region consisting of the *spaP*-*spaQ-spaR* genes (, S5, S6). The *groEL* amino acid dataset was aligned using ClustalW algorithm in BioEdit with default parameters [48] and all ambiguously aligned sites were removed from the further analyses. To gain more precise phylogenetic resolution within the *Sodalis* branch, we narrowed the taxon sampling and prepared an additional matrix using nucleotide sequences for the *groEL* gene. The matrix was aligned as described above and edited manually. The 16 S rDNA and the *spaPQR* dataset were aligned in the Mafft program [49], using the E-INS-i strategy with default parameters and manually edited in BioEdit [48]. For 16 S rDNA, the ambiguously aligned positions were eliminated in Gblocks [50].

Two approaches were used to infer phylogenetic trees, maximum likelihood (ML) and Bayesian inference (BI). For ML we used PhyML v3.0 [51], [52] with the SPR search algorithm. BI was performed in MrBayes 3.1.2 [53], [54] with five million generations and tree sampling every 100 generations. AWTY [55] was used to check the MCMC convergence and determine burn-in. Evolutionary substitution models for proteins and DNA were selected by ProtTest 2.4 [56] and for DNA by jModelTest 0.1.1 [57], respectively. For DNA sequences, the General Time Reversible (GTR) model was used with an estimated proportion of invariable sites (I) and heterogeneity of evolutionary rates modeled by the eight substitution rate categories of the gamma (Γ) distribution and the gamma shape parameter (alpha) estimated from the data. LG+I+Γ was determined as the best fitting model for the amino acid *groEL* dataset, and it was used for the ML analyses. Since this model is not implemented in MrBayes, we replaced it with WAG+I+Γ for the BI analysis. JTT+I+Γ model was used for the *spaPQR* concatenate in both BI and ML analyses.

### Cultivation of *Candidatus* Sodalis Melophagi sp. nov

The insects were surface sterilized using 96% ethanol and heamolymph was collected into a 1.5 ml eppendorf tube containing 500 μl of Mitsuhashi-Maramorosch (MM) medium [58] with 20% heat inactivated foetal bovine serum (FBS). The tube was incubated overnight at 27°C without shaking, a slightly modified protocol of Matthew et al. [25] was followed. Liquid culture was plated onto 10% sheep blood MMI plates solidified by 1% agar. The medium was supplemented with 100 μg/ml Polymyxin B and 10 mg/ml Amphotericin B to prevent contamination by Gram-negative non-symbiotic bacteria [59] and fungi. The plates were incubated at 27°C in a microaerobic atmosphere generated by the Campygen pack system (Oxoid) producing 5% of oxygen balanced with carbon dioxide. Bacteria were further inoculated into flasks containing C6/36 mosquito cells [60] (LGC Standards, Czech Republic) in MM medium with 20% heat inactivated FBS.

### Microscopy

A three day old liquid culture of C6/36 cells infected with *Candidatus* Sodalis melophagi was used for microscopic examination. Cells were harvested and fixed in 4% formaldehyde in PBS and observed under the BX53 microscope (Olympus) using Nomarski contrast. For electron microscopy, the midgut region containing bacteriome was dissected directly into a 2.5% glutaraldehyde in 0.1 M phosphate buffer and prefixed at 4°C overnight. The tissue was then postfixed at 4°C for 60 min with 2% osmium tetroxide in phosphate buffer. After dehydration through ethanol series, the samples were embedded in Spurr resin. Ultrathin sections were stained with uranyl acetate and lead citrate and examined in transmission electron microscope JEOL JEM-1010.

## Supporting Information

Figure S1 **16 S rDNA tree derived by ML method in PhyML.** Bootstrap values are indicated by the numbers at the nodes. New *Sodalis* lineages added in this study are printed in red. Values under 50 are not shown.
(EPS)

Figure S2 **Phylogenetic trees derived from** ***groEL*** **aminoacid matrix under ML in PhyML.** Bootstrap values are indicated by the numbers at the nodes. New *Sodalis* lineages added in this study are printed in red. Values under 50 are not shown.
(EPS)

Table S1 **List of sequences acquired in this study.**
(DOC)

Table S2 **Characteristics of particular datasets.**
(DOC)

Table S3 **List of primers used in this study.**
(DOC)

Table S4 **List of 16 S rDNA sequences used for phylogenetic inference.**
(DOC)

Table S5 **List of** ***groEL*** **sequences used for phylogenetic inference.**
(DOC)

Table S6 **List of** ***spaPQR*** **sequences used for phylogenetic inference.**
(DOC)
