# Supplementary material for: Candidatus Sodalis melophagi sp. nov.: Phylogenetically Independent Comparative Model to the Tsetse Fly Symbiont Sodalis glossinidius
Source: PLoS One. 2012 Jul 17;7(7):e40354. doi: 10.1371/journal.pone.0040354 (PMC3398932; doi:10.1371/journal.pone.0040354)
Supplement: Table S1 — List of sequences acquired in this study. (DOC) [file pone.0040354.s003.doc]

**Table S1: List of sequences acquired in this study.**

| Species | Specifity | Accession number |
| --- | --- | --- |
| *Biostraticola tofi* | *groEL* | JN865212 |
| Endosymbiont E1 of *Ornithomya avicularia* | 16S rDNA | JN872639 |
| Endosymbiont E2 of *Ornithomya avicularia* | 16S rDNA | JN872640 |
| Endosymbiont of *Rhampus pulicarius* | 16S rDNA | JN872638 |
| Endosymbiont of *Rhampus pulicarius* | *groEL* | JN900240 |
| *Candidatus* Sodalis melophagi | *groEL* | JN865213 |
| *Candidatus* Sodalis melophagi | 16S rDNA | JN872637 |
| *Candidatus* Sodalis melophagi | SSR-2 | JQ003581 |
| *Candidatus* Sodalis melophagi | SSR-3 | JQ003582 |
