# Supplementary material for: Candidatus Sodalis melophagi sp. nov.: Phylogenetically Independent Comparative Model to the Tsetse Fly Symbiont Sodalis glossinidius
Source: PLoS One. 2012 Jul 17;7(7):e40354. doi: 10.1371/journal.pone.0040354 (PMC3398932; doi:10.1371/journal.pone.0040354)
Supplement: Table S2 — Characteristics of particular datasets. (DOC) [file pone.0040354.s004.doc]

**Table S2: Characteristics of particular datasets**

| Dataset | Number of sites | Constant sites | Uninformative sites | Informative sites |
| --- | --- | --- | --- | --- |
| *groEL* nucleotides | 1614 | 1006 | 209 | 339 |
| *groEL* aminoacids | 518 | 271 | 77 | 170 |
| 16S rDNA | 1405 | 995 | 176 | 234 |
| *spaPQR* | 429 | 213 | 120 | 96 |
