# Supplementary material for: Candidatus Sodalis melophagi sp. nov.: Phylogenetically Independent Comparative Model to the Tsetse Fly Symbiont Sodalis glossinidius
Source: PLoS One. 2012 Jul 17;7(7):e40354. doi: 10.1371/journal.pone.0040354 (PMC3398932; doi:10.1371/journal.pone.0040354)
Supplement: Table S3 — List of primers used in this study. (DOC) [file pone.0040354.s005.doc]

**Table S3: List of primers used in this study.**

| Primer name | Specifity | Sequence | Reference |
| --- | --- | --- | --- |
| SODGroELf | *groEL Sodalis* specific | CCA AAG CTA TCG CTC AGG TAG G | [1] |
| SODGroELr | *groEL Sodalis* specific | TTC TTT GCC CAC TTT CGC CAT A | [1] |
| GroELforward | *groEL* enterobacteria | GCT AAA GAI GTI AAR TTY GG | This study |
| GroELreverse | *groEL* enterobacteria | TTA CAT CAT ICC ICC CAT ICC | This study |
| 16S forward | 16S rDNA eubacterial | GCT TAA CAC ATG CAA G | [2] |
| 16S reverse | 16S rDNA eubacterial | CCA TTG TAG CAC GTG T | [2] |
| F40 forward | 16S rDNA enterobacteria | GCG GCA AGC CTA ACA CAT | [3] |
| R1060 reverse | 16S rDNA enterobacteria | CTT AAC CCA ACA TTT CTC AAC ACG AG | [3] |
| SoMelSSR2_F2 | SSR-2 assembly | GCA TGC CCT GGG CGT TCC ATG | This study |
| SoMelSSR2_R2 | SSR-2 assembly | CCA TGC GCC CGA TCT GCT CAA | This study |
| SoMelSSR3_F2 | SSR-3 assembly | CCG GTA CGC TAC TGC AAA TTG | This study |
| SoMelSSR3_R2 | SSR-3 assembly | GGG CAT GCG CTC AGC ATC GA | This study |

**Supplement references**

1. Matthew CZ, Darby AC, Young SA, Hume LH, Welburn SC (2005) The rapid isolation and growth dynamics of the tsetse symbiont Sodalis glossinidius. FEMS Microbiololgy Letters 248: 69-74.

2. O'Neill SL, Giordano R, Colbert AM, Karr TL, Robertson HM (1992) 16S rRNA phylogenetic analysis of the bacterial endosymbionts associated with cytoplasmic incompatibility in insects. Proceedings of the National Academy of Sciences of the United States of America 89: 2699-2702.

3. Hypša V, Křížek J (2007) Molecular evidence for polyphyletic origin of the primary symbionts of sucking lice (Phthiraptera, Anoplura). Microbial ecology 54: 242-251.
