# Supplementary material for: Candidatus Sodalis melophagi sp. nov.: Phylogenetically Independent Comparative Model to the Tsetse Fly Symbiont Sodalis glossinidius
Source: PLoS One. 2012 Jul 17;7(7):e40354. doi: 10.1371/journal.pone.0040354 (PMC3398932; doi:10.1371/journal.pone.0040354)
Supplement: Table S4 — List of 16 S rDNA sequences used for phylogenetic inference. (DOC) [file pone.0040354.s006.doc]

**Table S4: List of 16S rDNA sequences used for phylogenetic inference.**

| Species | Accession number | Species | Accession number |
| --- | --- | --- | --- |
| *Biostraticola tofi* | AM774412 | Endosymbiont of *Puto* sp. | DQ133565 |
| *Brenneria quercina* | HM196339 | Endosymbiont of *Puto yuccae* | HM449987 |
| *Edwardsiella ictaluri* | NC_012779 | Endosymbiont of *Rhampus pulicarius* | JN872638 |
| *Edwardsiella tarda* | NC_013508 | Endosymbiont of *Sitophilus granarius* | AY126638 |
| Endosymbiont *Archarius roelofsi* | AB604872 | Endosymbiont of *Sitophilus oryzae* | AF005235 |
| Endosymbiont E1 of *Ornithomya avicularia* | JN872639 | Endosymbiont of *Sitophilus rugicollis* | AY126639 |
| Endosymbiont E2 of *Ornithomya avicularia* | JN872640 | Endosymbiont of *Sitophilus zeamais* | M85269 |
| Endosymbiont of *Cantao ocellatus* | AB541010 | Endosymbiont of *Tetropium castaneum* | AM946408 |
| Endosymbiont of *Columbicola columbae* str. BNS06 | AB303386 | *Gibbsiella quercinecans* | GU562337 |
| Endosymbiont of *Columbicola columbae* str. BRB07 | AB303387 | *Photorhabdus asymbiotica* | NC_012962 |
| Endosymbiont of *Columbicola columbae* str. FKK99 | AB303383 | *Photorhabdus luminescens* | NC_005126 |
| Endosymbiont of *Columbicola columbae* str. SMY06 | AB303384 | *Proteus mirabilis* | NC_010554 |
| Endosymbiont of *Columbicola columbae* str. SPR06 | AB303382 | *Serratia rubidaea* | HM585373 |
| Endosymbiont of *Columbicola columbae* str. TTR06 | AB303385 | *Sodalis glossinidius* ex *Glossina austeni* | U64869 |
| Endosymbiont of *Craterina melbae* | EF174495 | *Sodalis glossinidius* ex *Glossina brevipalpis* | U64870 |
| Endosymbiont of *Curculio hachijoensis* | AB604873 | *Sodalis glossinidius* ex *Glossina fuscipes* | U64868 |
| Endosymbiont of *Curculio sikkimensis* AB507712 | AB507712 | *Sodalis glossinidius* ex *Glossina morsitans* | NC_007712 |
| Endosymbiont of *Curculio sikkimensis* AB514505 | AB514505 | *Sodalis glossinidius* ex *Glossina pallidipes* | M99060 |
| Endosymbiont of *Curculio sikkimensis* AB517595 | AB517595 | *Sodalis glossinidius* ex *Glossina palpalis* | U64867 |
| Endosymbiont of *Eucorysses grandis* | AB571330 | *Candidatus* Sodalis melophagi | JN872637 |
| Endosymbiont of *Puto albicans* HM449985 | HM449985 | *Vibrio cholerae* | NC_002505 |
| Endosymbiont of *Puto albicans* HM449984 | HM449984 | *Xenorhabdus bovienii* | NC_013892 |
| Endosymbiont of *Puto barberi* | HM449983 | *Xenorhabdus nematiophila* | NC_014228 |
