# Supplementary material for: Candidatus Sodalis melophagi sp. nov.: Phylogenetically Independent Comparative Model to the Tsetse Fly Symbiont Sodalis glossinidius
Source: PLoS One. 2012 Jul 17;7(7):e40354. doi: 10.1371/journal.pone.0040354 (PMC3398932; doi:10.1371/journal.pone.0040354)
Supplement: Table S5 — List of groEL sequences used for phylogenetic inference. (DOC) [file pone.0040354.s007.doc]

**Table S5: List of *groEL* sequences used for phylogenetic inference.**

| Species | Accession number | Species | Accession number |
| --- | --- | --- | --- |
| *Arsenophonus nasoniae* | FN545234 | *Ishikawaella capsulata* | JF736508 |
| *Arsenophonus triatominarum* | DQ508202 | *Klebsiella planticola* | AB008148 |
| *Baumannia cicadellinicola* | NC007984 | *Klebsiella pneumoniae* | NC012731 |
| *Biostraticola tofi* | JN865212 | *Kleidoceria schneideri* | FN555108 |
| *Blochmannia floridanus* | NC005061 | *Moranella endobia* | NC015735 |
| *Blochmannia pennsylvanicus* | NC007292 | *Pantoea ananatis* | NC013956 |
| *Buchnera aphidicola* APS | BA000003 | *Pantoea vagans* | NC014562 |
| *Buchnera aphidicola* BP | AE016826 | *Pectobacterium carotovorum* | NZABVY01000132 |
| *Citrobacter koseri* | NC009792 | *Pectobacterium wasabiae* | NC013421 |
| *Citrobacter rodentium* | NC013716 | *Photorhabdus asymbiotica* | FM162591 |
| *Cronobacter sakazaki* | NC009778 | *Photorhabdus luminescens* | BX571872 |
| *Cronobacter turicensis* | NC013282 | *Proteus mirabilis* | NZACLE01000068 |
| *Curculioniphilus buchneri* | AB514500 | *Pseudomonas aeruginosa* | CP000744 |
| *Dickeya dadantii* | NC013592 | *Puchtella pedicinophila* | AB478980 |
| *Dickeya zeae* | NC012912 | *Rahnella* sp. | NC015061 |
| *Edwardsiella ictulari* | NC012779 | *Riesia pediculicola* | CP001085 |
| *Edwardsiella tarda* | NZADGK01000011 | *Rohrkolberia cinguli* | FR729476 |
| Endosymbiont of antlion Kuo1-1 strain | AB375448 | *Salmonella enterica* | NZ_CAAU01001059 |
| Endosymbiont of antlion Kuo2-2 strain | AB375454 | *Serratia marcescens* | AB008145 |
| Endosymbiont of *Cantao ocellatus* | AB541012 | *Serratia symbiotica* | NZGL636112 |
| Endosymbiont of *Curculio sikkimensis* | AB507719 | *Shigella flexneri* | NC008258 |
| Endosymbiont of *Rhampus pulicarius* | JN900240 | *Sodalis glossinidius* | NC007712 |
| Endosymbiont of *Sitophilus oryzae* | AF005236 | *Candidatus* Sodalis melophagi | JN865213 |
| *Erwinia amylovora* | NC013961 | *Wigglesworthia glossinidia* | NC004344 |
| *Erwinia pyrifoliae* | NC012214 | *Xanthomonas campestris* | AE008922 |
| *Erwinia tasmaniensis* | NC010694 | *Xenorhabdus bovienii* | FN667741 |
| *Escherichia coli* | AFAT01000088 | *Xenorhabdus nematophila* | AY184491 |
