# Supplementary material for: Candidatus Sodalis melophagi sp. nov.: Phylogenetically Independent Comparative Model to the Tsetse Fly Symbiont Sodalis glossinidius
Source: PLoS One. 2012 Jul 17;7(7):e40354. doi: 10.1371/journal.pone.0040354 (PMC3398932; doi:10.1371/journal.pone.0040354)
Supplement: Table S6 — List of spaPQR sequences used for phylogenetic inference. (DOC) [file pone.0040354.s008.doc]

**Table S6: List of *spaPQR* sequences used for phylogenetic inference.**

| Species | Accession number (*spaP*, *spaQ*, *spaR*) |
| --- | --- |
| *Chromobacterium violaceum* | NP_902294, NP_902293, NP_902292 |
| Endosymbiont of *Craterina melbae* | EF174496 |
| Endosymbiont of *Sitophilus zeamais* (SZPE) | AF426456 |
| *Salmonella enterica* | NP_457284, NP_457283, NP_457282 |
| *Sodalis glossinidius* (Toh et al. 2006) | YP_455764, YP_455763, YP_455762 |
| *Sodalis glossinidius* (Dale et al. 2001) | AF306650 |
| *Candidatus* Sodalis melophagi | JQ003581 |
